# Validation and reliability of the Tracheostoma Well-Being Score (TWBS) in critically ill patients

**DOI:** 10.1007/s00068-025-02948-x

**Published:** 2025-08-22

**Authors:** Christopher Ull, Alexander Heimes, Uwe Hamsen, Oliver Cruciger, Aileen Spieckermann, Thomas Armin Schildhauer, Robert Gaschler, Christian Waydhas, Christina Ingwald

**Affiliations:** 1 Department of General and Trauma Surgery, BG University Hospital Bergmannsheil, Bürkle-de-la-Camp-Platz 1, 44789 Bochum, Germany; 2 Faculty of Psychology, FernUniversität of Hagen, Universitätsstraße 47, 58097 Hagen, Germany; 3 Department of Trauma Surgery, University Hospital Essen, Hufelandstr. 55, 45147 Essen, Germany; 4 Department of General and Trauma Surgery, BG University Hospital Bergmannsheil, North Rhine-Westphalia), Bürkle-de-la-Camp-Platz 1, 44789 Bochum, Germany

**Keywords:** Tracheostomy, Intensive care unit, Tracheostomy well-being score, Well-being assessment in tracheotomized patients

## Abstract

**Objectives:**

Validation and Reliability of the Tracheostoma Well-Being Score (TWBS) in critically ill patients.

**Design:**

Prospective observational study involving tracheotomized patients of a tertiary university referring center.

**Method:**

Sixty-five tracheotomized patients completed the TWBS on at least two or more days. External assessments of probable problems with the inserted cannula for each patient were provided by the nurse treating that patient on the respective day.

**Results:**

The test-retest reliability demonstrated a stable response pattern over repeated measurements. Criterion validity revealed a limited agreement between external assessment by healthcare providers and patients’ self-reported experience, highlighting discrepancies in symptom perception.

**Conclusion:**

The 12-Item-TWBS appears to be reliable. The results further underline the necessity of using a validated self-reporting tool to assess patient comfort, as external assessment appears to have shortcomings. The TWBS might be a valuable tool to more accurately appraise patient comfort with the indwelling tracheostomy cannula.

## Introduction

In cases of critical illness, an airway intervention is often necessary to clear the airway or ensure effective mechanical ventilation (MV) [1]. The increasing number of tracheostomies is driven by respiratory failure, infective cough, neurological injury, and carcinoma, which have become more prevalent in older people [2]. Approximately 5–10% of intensive care unit (ICU) patients undergo tracheostomy [3], with up to 34% requiring mechanical ventilation for more than 48 h [4]. A tracheostomy significantly impacts the psychological well-being and social interactions of those living with it [1]. Several studies reported that communication challenges are a major source of distress for patients undergoing laryngectomy or tracheostomy due to neck and head cancer. These difficulties frequently lead to anxiety, depression and/or frustration and other psychological issues in the affected patients [5, 6]. Critically ill patients on the ICU with an inserted cannula reported experiencing limited communication abilities, persistent coughing or fear. Limited research has been conducted on high-quality care of patients following on tracheostomy and it has been suggested that future studies should address this gap [7]. The *Tracheostomy Well-Being Score* (TWBS) provides an assessment of patient well-being while the cannula is still in place and allows to identify relevant factors influencing their experience and quality of life at an early stage and to develop targeted interventions accordingly [8]. The initial 25-item questionnaire covering six categories (respiration, coughing, pain, speaking, swallowing, and comfort) was refined into a 12-item TWBS for clinical use.

The aim of the present study was to validate the TWBS for standard use in the intensive care setting. To achieve this, the 12-item questionnaire was administered at a minimum of two measurement time points in the ICU to evaluate its applicability and reliability in this specific setting.

## Methods

### Study design

**Fig. 1:**
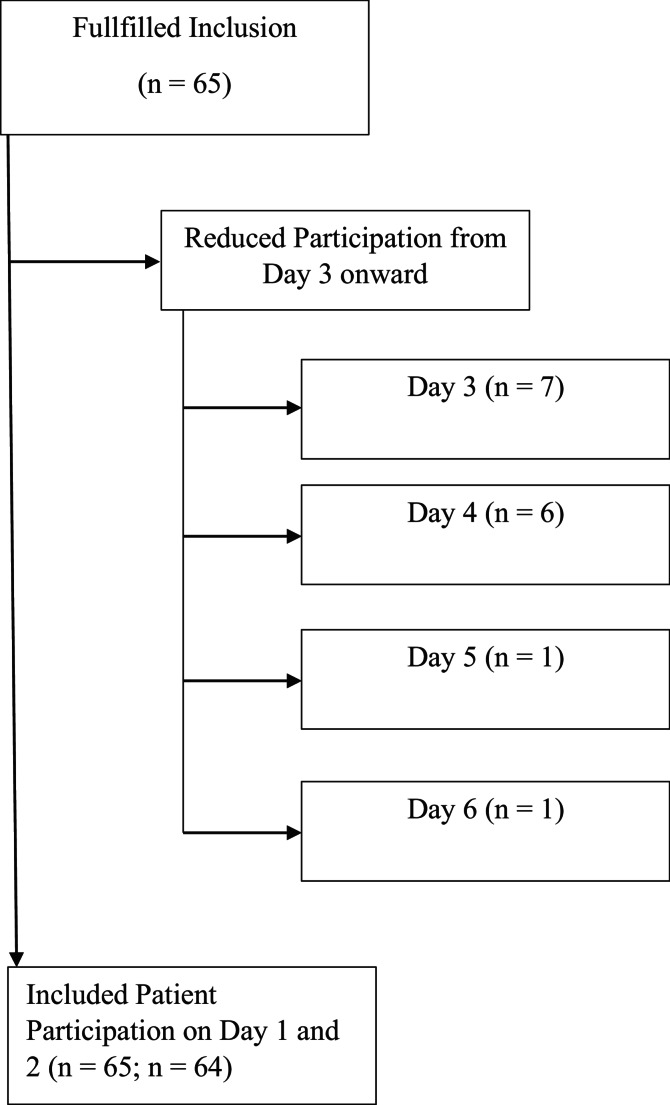
Flow diagram showing patient search

This prospective, single center, observational study was performed at one surgical ICU, one intermediate care (IMC) and one ward for spinal cord injured patients of the BG University Hospital Bergmannsheil Bochum. Patients were prospectively surveyed from 23.05.2023 to 18.07.2024. All patients from the three units were included during the study period if they met al.l of the following criteria: (1) age ≥ 18 years, (2) tracheotomized for more than 48 h and expected to remain cannulated at least for the next 7 days, (3) adequate cognitive and motoric skills to answer the survey manually and/or verbally, (4) a score of −1, 0, + 1 points on the Richmond Agitation-Sedation Scale (RASS) [9] or a score of < 3 points on the nursing delirium scale (Nu-DESC) [10] in individuals without sedation, (5) successful completion of the questionnaire for at least two or more days. No participants were excluded from the study; therefore, data from *N* = 65 on day 1 and *N* = 64 on day 2 were analyzed (Fig. 1).

### Standardized assessment

Prior to inclusion and to each session, the patient´s level of consciousness was assessed using the RASS and the Nu-DESC. The TWBS includes six categories (respiration, coughing, pain, speaking, swallow and comfort) with two items each. The items are to be answered on a 4-point Likert scale (0 = never, 1 = rarely, 2 = often, 3 = always). The items per category can be taken from Table 2 in the Appendix. Patients were recruited consecutively from three specialized units. External assessments were conducted by a medical student and additionally trained paramedic (AH), who interviewed the patient’s primary nurse on each assessment day. This approach reflects a single-rater design, as only the nurse responsible for the patient on that specific day was consulted. Ratings were provided using a 4-point Likert scale (0 = unlikely, 1 = moderate, 2 = likely, 3 = quite likely). The procedure was verbally explained to the persons and informed content was obtained in written form, or if not possible, verbally via head nodding, or by blinking. This was confirmed by persons who were unrelated to the investigation. Depending on their current fine motor abilities and communication skills, patients completed the 12-item survey either manually using paper and pen or verbally, with the experimental supervisor reading the questions aloud. The survey was repeated after two to four days.

### Statistical analysis

A priori power analysis (correlation: point biserial model) performed with G*Power 3.1 [11] required a sample size of *n* = 64, for a power of 0.80 with an alpha-error of 0.05 and an average effect size of *r* =.3. Statistical analysis was performed using R-Studio (Version 2024.09.1). Demographic data are presented as mean and standard deviation or absolute numbers and percentage. An independent t-test was conducted to identify potential differences in perception between patients with tetraplegia those who underwent tracheostomy for other reasons (applicability to different patient populations). T-tests were performed for the categories breathing, pain and comfort. Test-retest reliability was assessed via correlation of individual patient profiles over repeated measurements. Criterion validity was assessed with the Pearson-correlation coefficient. The significance level was set to α = 0.05.

## Results

### Demographic and intensive care data

The demographic data of the 65 enrolled patients are listed in detail in Table 1 in the Appendix. The included patients were nine females (13.8%) and 56 males (86.2%) with a mean age of 56.85 ± 16.23. The main diagnoses for ICU admission were spinal cord injuries, followed by other trauma surgery diagnoses (see also Table 1). The majority of the patients (80%) were able to answer the questionnaire verbally. The remaining patients answered the questions via head nodding after reading the item aloud via the examiner. Fifteen patients (23.1%) were on long-term mechanical ventilation. All of the patients could be surveyed twice, a third and a fourth survey round could be achieved for 7 and 6 patients, respectively. Due to the decreasing number of participants, we only used the data from the first two survey rounds.

### Applicability to different patient populations

An independent t-test was conducted to examine potential differences at the group level (Tracheostoma in patients with tetraplegia [M_1_] vs. tracheostomy for other reasons[M_2_]) in the categories breathing, pain and comfort. Overall, no significant group differences were found: Breathing (*M*_*1*_ = 1.40, *SD*_*1*_ = 0.78; *M*_*2*_ = 1.52, *SD*_*2*_ = 0.57; *t*(63) = − 0.676, *p* =.501), pain (*M*_*1*_ = 1.08, *SD*_*1*_ = 0.83; *M*_*2*_ = 1.48, *SD*_*2*_ = 0.73; *t*(63) = −1.891, *p* =.063), comfort (*M*_*1*_ = 0.72, *SD*_*1*_ = 0.52; *M*_*2*_ = 0.86, *SD*_*2*_ = 0.56; *t*(63) = −1.026, *p* =.309).

### Retest-reliability

In order to test whether the six subscores together capture a reliable profile that can characterize the condition of a patient, we assessed profile stability. To this end we computed for each participant a correlation between the six scores for day 1 and the six scores for day 2. The median of these correlations was 0.895. The mean (z-transformed, averaged, re-transformed) was 0.859 (*t*(63) = 14.16, *p* <.001) underlining that we indeed captured a reliable profile based on the six subsocres.

### Criterion validity

To assess the level of agreement between patient self-reports and clinician ratings, a criterion-based correlation analysis was conducted. This approach was chosen to determine whether patients’ subjective experiences align with external clinical assessments. The results (Table 3) indicate that the overall agreement was low, with only a few significant correlations observed. Notably, significant positive correlations were found for respiration ratings on both days (Day 1: *r* =.265, *p* =.034; Day 2: *r* =.285, *p* =.022). Additionally, the assessment of coughing showed a significant correlation on Day 2 (*r* =.278, *p* =.028). Pain ratings demonstrated a moderate positive correlation on Day 1 (*r* =.292, *p* =.019), though this was not replicated on Day 2. However, the majority of symptom categories showed little to no correlation between self-reports and external assessment, indicating fundamental discrepancy in how symptoms are perceived by patients versus healthcare providers. This suggests a limited overlap between patient-reported outcomes and clinician evaluations, highlighting potential discrepancies in symptom perception between both perspectives.

## Discussion

When selecting patients for tracheostomy it is crucial to prioritize patient-centered outcomes [12]. The presence of a tracheostomy complicates communication, as it often limits verbal speech and requires alternative communication methods. With the TWBS a standardized instrument is given to assess patients comfort following a tracheostomy on a regular basis. The results indicate a high test-retest validity, suggesting consistent patient ratings over time, while criterion validity analysis revealed only low agreement between patient-reported outcomes and clinician assessment, highlighting potential discrepancy in symptom perception.

Spinal cord injuries or other neurological conditions like amyotrophic lateral sclerosis (ALS) have fundamental impact on communication skills. Depending on the severity of the diagnosis and the extent of symptoms, communication may be significantly impaired or even completely absent. Research indicates that individuals with communication impairments are more likely to receive lower-quality care and face higher risks of preventable adverse events [13]. It is important to emphasize that these results do not contradict the general assumption that communication impairments can affect the quality of care. Rather, this could be due to the fact that all studied patients were treated in a standardized intensive care or a highly specialized environment, where structural conditions may migitate potential differences. Moreover, our study focused on specific aspects such as breathing, pain, and comfort, rather than other indicators of care quality or the occurrence of adverse events. While our results suggest that the perception of breathing, pain and comfort is not systematically influenced by the underlying condition leading to tracheostomy, one might have expected different perception profiles between patients between with and without tetraplegia, given the distinct physiological and neurological characteristics. Studies indicate, that individuals with tetraplegia frequently exhibit altered cough reflexes and impaired secretion management, which may affect their perception of airway discomfort and suction needs [14].

Istanboulian and colleagues [15] pointed out that identifying, implementing, and sustaining standard augmented and alternative communication (AAC) practices for communication with non-speaking patients still presents a complex challenge to person-centered care due to persistent barriers. However, it has been demonstrated, that AAC can be integrated into the care of such patients [5, 8, 16]. The 12-item TWBS scale demonstrates that patient-centered care can be effectively implemented in complex settings, both with and without AAC. Due to its brevity, the questionnaire can be easily administered verbally by nursing staff, ensuring seamless incorporation into daily routines. Furthermore, when deployed on an AAC device, such an eyetracker, it remains simple to integrate into daily workflow, enhancing communication and care delivery in the ICU.

The low overall agreement between patient self-reports and clinician ratings, with only a few significant correlations, suggests limited overlap between these perspectives. This discrepancy aligns with existing literature indicating that patients often report symptom frequency and severity earlier than clinicians, and their assessments are more predictive of daily health status, whereas clinicians’ evaluations are more related to clinical outcomes [17, 18]. Patients may prioritize subjective experiences and daily functioning, while clinicians might emphasize observable clinical signs and objective measures. This divergence can lead to variations in reported outcomes [18]. Additionally, individual differences, such as health literacy, cognitive function, and personal expectations, can influence self-reported outcomes. Factors like comprehension of symptoms and recall ability may affect patients’ responses, leading to discrepancies between patient and clinician assessments [19].

Finally, the observed stability of individual subscores between Day 1 and Day 2 indicates a consistent profile over time, suggesting that patients’ responses are consistent over time. This consistency underscores the instrument’s stability and its potential utility in both clinical and research settings.

## Conclusion

The Tracheostomy Well-Being Scale (TWBS) provides a standardized instrument to assess patients’ comfort following a tracheostomy on a regular basis, enabling continuous monitoring of their status. Regular use of the TWBS can help identify the need for suctioning or cannula changes in a timely manner, thereby improving both quality of care and subjective well-being of tracheotomized patients. Our results indicate that the perception of breathing, pain, and fit comfort is not systematically influenced by the underlying condition leading to tracheotomy, suggesting that the instrument captures similar experiences across different patient groups. While this may imply a lack of discriminant validity, it also supports the TWBS’s general applicability across various patient populations. Furthermore, our findings emphasize the importance of patient-centered outcomes when selecting candidates for tracheostomy [12], especially given the significant impact on communication that tracheostomies can have. In sum, our study underlines the necessity that the 12-item-TWBS should be used as standard for tracheostomized patients in order to provide them with the most comfortable possible comfort with the insertion of the tracheostomy cannula.

### Limitation

It would have been preferable to survey more patients at multiple testing time points; however, this was often not feasible, as patients were transferred to other wards or unavailable at scheduled testing times due to further examinations. More subtle differences among patient populations might have been detectable with an even larger sample size. Further work might follow up on individual factors such as health literacy and cognitive impairments that potentially influence the self-report. Specifically, we acknowledge that calculating test-retest reliability based on only two measurement points may overestimate the results and that more time points in future studies would allow for a more differentiated understanding of temporal stability.

## Data Availability

The datasets used and/or analyzed during the current study are available from the corresponding author on reasonable request.

## Appendix

**Table 1:** Demographic and intensive care data of enrolled patients (N = 65)

Female-to-male ratio	9 (13.8%) : 56 (86.2%)
Age (years)	56.85 ± 16.23
*Main Diagnoses*	
Spinal Cord Injuries	45 (69.2%)
Trauma Surgery	18 (27.7%)
Neurotraumatology	2 (3.1%)
*Technique of tracheostomy*	
Surgical	22 (33.8%)
Percutaneous dilatational	43 (66.2%)
Acute tracheostomy	50 (76.9%)
Chronic tracheostomy (long-term)	15 (23.1%)
*Ventilation mode*	
Pressure-support ventilation	14 (21.5%)
Volume-controlled ventilation	18 (27.7%)
Spontaneous-breathing trial	33 (50.8%)
Time from tracheostomy to testing (days)	530.14 ± 1673.33
*Time between Testing Days (days)*	
Day 1 to Day 2	4.42 ± 8.82
Day 2 to Day 3	6.43 ± 27.62
Day 3 to Day 4	2.50 ± 0.82

Note. Data presented as absolute numbers (percentage) or mean ± standard deviation

**Table 2:** The 12 items of the tracheostomy well-being score

Category	Items
*Respiration*	How often do you experience shortness of breath?
	How often do you feel that your trachea is dry?
*Coughing*	How often do you cough?
	Do you have coughing attacks while you speak?
*Pain*	How often do you have pain while suctioning?
	How often do you have pain when changing your cannula?
*Speaking*	How often do you think you are well understood with whispered speech?
	How often do you feel the volume of your speech is normal?
*Swallowing*	How often does your tracheostomy tube interfere with swallowing liquids or food?
	How often do you feel saliva flowing into the trachea?
*Comfort*	How often do you feel shame or disgust because of your tracheostomy tube?
	How often do you find the fixation of your tracheostomy tube comfortable?

Note. The items are to be answered on a 4-point Likert scale (0 = never, 1 = rarely, 2 = often, 3 = always)

**Table 3:** Criterion validity – pearson correlation values for the rating between self-reports and clinician rating of the respective category

*Category*	Day one		Day two		
	*r*	*p*	*r*	*p*	
Respiration:	0.265	0.034*	0.285	0.022*	
Coughing:	0.013	0.922	0.278	0.028*	
Pain:	0.292	0.019*	− 0.015	0.909	
Speaking:	− 0.192	0.182	− 0.140	0.270	
Swallowing:	0.218	0.083	0.123	0.332	
Comfort:	0.204	0.107	0.079	0.541	

*Note. *p <.05*
